# Role of gut microbiota in regulating immune checkpoint inhibitor therapy for glioblastoma

**DOI:** 10.3389/fimmu.2024.1401967

**Published:** 2024-06-10

**Authors:** Hao Zhang, Ying Hong, Tingting Wu, Eyi Ben, Shuai Li, Liu Hu, Tao Xie

**Affiliations:** ^1^ Department of Radiation Oncology, Hubei Cancer Hospital, TongJi Medical College, Huazhong University of Science and Technology, Wuhan, Hubei, China; ^2^ Department of Health Management, Hubei Cancer Hospital, TongJi Medical College, Huazhong University of Science and Technology, Wuhan, Hubei, China; ^3^ Department of Oncology, Yidu People’s Hospital, Yichang, Hubei, China; ^4^ Department of Urology, The First Affiliated Hospital of Zhengzhou University, Zhengzhou, Henan, China

**Keywords:** glioblastoma, gut microbiota, immunotherapy, immune checkpoint inhibitor, gut-brain axis

## Abstract

Glioblastoma (GBM) is a highly malignant, invasive, and poorly prognosed brain tumor. Unfortunately, active comprehensive treatment does not significantly prolong patient survival. With the deepening of research, it has been found that gut microbiota plays a certain role in GBM, and can directly or indirectly affect the efficacy of immune checkpoint inhibitors (ICIs) in various ways. (1) The metabolites produced by gut microbiota directly affect the host’s immune homeostasis, and these metabolites can affect the function and distribution of immune cells, promote or inhibit inflammatory responses, affect the phenotype, angiogenesis, inflammatory response, and immune cell infiltration of GBM cells, thereby affecting the effectiveness of ICIs. (2) Some members of the gut microbiota may reverse T cell function inhibition, increase T cell anti-tumor activity, and ultimately improve the efficacy of ICIs by targeting specific immunosuppressive metabolites and cytokines. (3) Some members of the gut microbiota directly participate in the metabolic process of drugs, which can degrade, transform, or produce metabolites, affecting the effective concentration and bioavailability of drugs. Optimizing the structure of the gut microbiota may help improve the efficacy of ICIs. (4) The gut microbiota can also regulate immune cell function and inflammatory status in the brain through gut brain axis communication, indirectly affecting the progression of GBM and the therapeutic response to ICIs. (5) Given the importance of gut microbiota for ICI therapy, researchers have begun exploring the use of fecal microbiota transplantation (FMT) to transplant healthy or optimized gut microbiota to GBM patients, in order to improve their immune status and enhance their response to ICI therapy. Preliminary studies suggest that FMT may enhance the efficacy of ICI therapy in some patients. In summary, gut microbiota plays a crucial role in regulating ICIs in GBM, and with a deeper understanding of the relationship between gut microbiota and tumor immunity, it is expected to develop more precise and effective personalized ICI therapy strategies for GBM, in order to improve patient prognosis.

## 1 Introduction

Glioblastoma (GBM) is a highly malignant tumor of the nervous system that occurs mainly in the glial cells of the brain. It has one of the highest incidence rates and is the most complicated to treat among all brain tumors. It grows rapidly, with a short disease course and poor prognosis (1). For GBM, surgery combined with radiotherapy and chemotherapy is commonly used as the standard treatment. However, even with active treatment, the median survival period is only 15 months (2). Studies have shown that immune checkpoint inhibitors (ICIs) play a role in GBM (3), and are expected to become a new treatment strategy.

ICIs can relieve the inhibitory state of the patient’s immune system and activate T cells to attack tumors, thereby controlling the growth and spread of the tumor (4). However, ICI therapy has certain limitations. For example, not all patients benefit from ICI therapy; some patients may not respond to treatment or develop resistance, and ICI therapy may cause adverse reactions, such as immune-related adverse reactions, fatigue, and nausea (5). Researchers have found that gut microbiota plays a crucial role in regulating ICI therapy in GBM (6). The relationship between gut microbiota and ICI therapy for GBM is a complex topic of research. Currently, studies are underway on how the gut microbiota affects ICI therapy for GBM, and how to utilize it to enhance the effectiveness of ICI therapy. Notably, the composition and diversity of gut microbiota may affect the function of the immune system, thereby affecting the efficacy of ICIs in GBM treatment. For example, metabolites produced by the gut microbiota, such as short-chain fatty acids (SCFAs), tryptophan (Trp), and arginine, may affect immune cell activity, thereby affecting the effectiveness of ICI therapy (7). In addition, the gut microbiota may affect the immunogenicity of tumor cells, namely their ability to recognize and attack the immune system, which can also affect the effectiveness of ICI therapy (8). We are currently exploring methods to utilize the gut microbiota to enhance the efficacy of ICIs in GBM treatment. Some studies have shown that the activity and function of immune cells can be improved by regulating the composition and diversity of the gut microbiota, thereby enhancing the effectiveness of ICI therapy (9).

Taken together, the relationship between the gut microbiota and ICI therapy for GBM is an emerging research field. Although there is currently no clear conclusion, it has broad research prospects. This article reviews the role of gut microbiota in regulating ICIs in GBM to provide new ideas and methods for GBM.

## 2 Relationship between the gut microbiota and GBM

The gut microbiota includes a large number of microorganisms that colonize the human gut, affecting the body’s digestive function, defense ability, susceptibility to autoimmune diseases and tumors, and the body’s response to disease-treating drugs (10). Probiotics can promote intestinal peristalsis and food digestion, inhibit the growth of pathogenic bacteria, decompose harmful substances, and enhance immunity. Neutral bacteria have a dual function and are harmless to the human body under normal circumstances. When the bacterial community system is disrupted, bacteria can proliferate in large quantities and cause diseases; pathogenic bacteria, in particular, are harmful to the human body and benefit from microbial constraints. Once their quantity increases significantly, they can affect the immune system and cause diseases (11).

**Figure 1:**
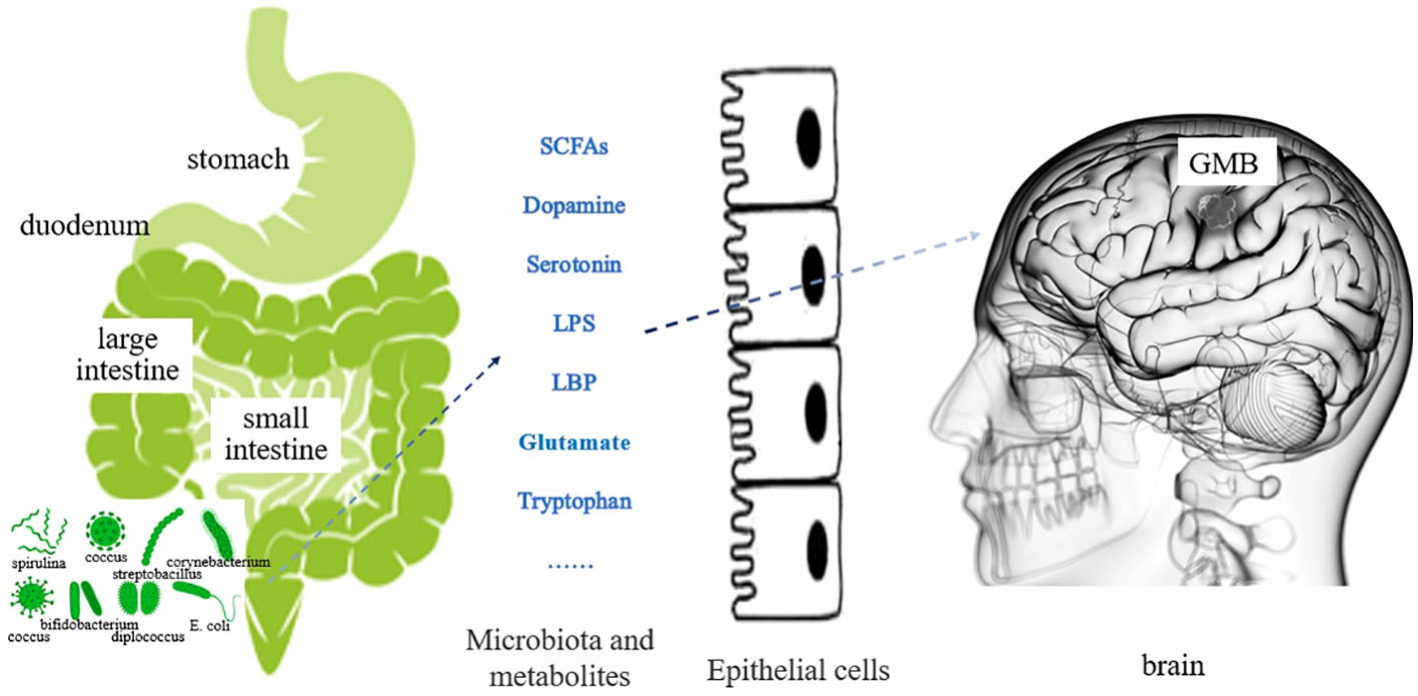
Effect of gut microbiota metabolites on GBM (The various metabolites of gut microbiota are secreted into the bloodstream through the intestinal mucosal epithelium. Regulating changes in GBM cells through blood circulation or affecting neurotransmitter function.).

**Figure 2:**
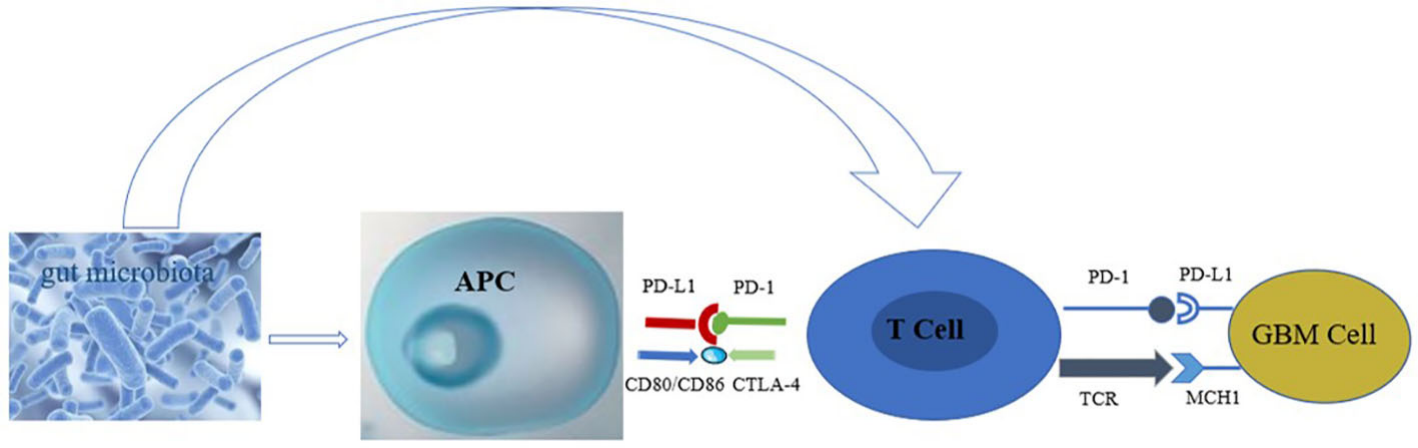
Effect of gut microbiota on immune cells in GBM (The gut microbiota stimulates the body to release antigens, which are taken up and processed by APC, presented to T cells, or directly acts on T cells, causing the PD-1/TCR receptors on the surface of T cells to bind to the corresponding ligands on the surface of GBM, inhibiting the proliferation of GBM cells).

**Figure 3:**
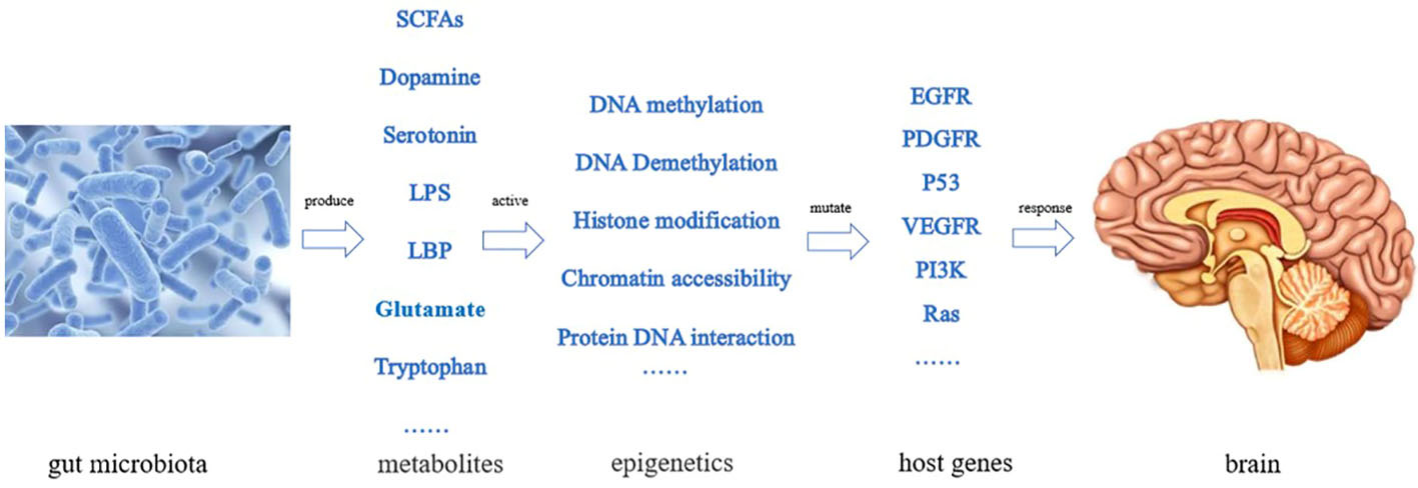
Effect of gut microbiota on GBM by affecting the expression of host genes (The gut microbiota produces various metabolic products, stimulating epigenetic changes in the body, leading to some gene mutations and causing proliferation and differentiation of GBM cells).

The gut microbiota plays a role in the growth and development of GBM (12), through various mechanisms. For example, the metabolites produced by intestinal bacteria promote the proliferation and migration of GBM cells and accelerate tumor growth and spread (13). Research has shown that in GBM, metabolites of gut microbiota such as 5-hydroxytryptamine, Norepinephrine, Glutamine, and lipopolysaccharide binding proteins (LBP) can promote the proliferation and migration of GBM cells, while dopamine, lipopolysaccharides (LPS) play the opposite role (14–16) (**Figure 1**). In addition, the composition and abundance of the gut microbiota can affect the activation and function of immune cells, thereby affecting the immune response to tumors. For example, dysbiosis of the gut microbiota can lead to immune system dysfunction, reduce the immune response to tumors, and promote tumor growth and progression (17) (**Figure 2**). Furthermore, the gut microbiota can influence GBM development by influencing the expression of host genes. Metabolites of the gut microbiota can interact with host cell genes, thereby affecting their expression and function. This can affect the proliferative, differentiative, and invasive abilities of GBM cells, affecting tumor development (18) (**Figure 3**). Understanding the relationship between gut microbiota and GBM will aid our understanding of the pathogenesis of GBM, which will help develop diagnostic and therapeutic strategies based on the gut microbiota to improve the therapeutic efficacy of GBM.

## 3 The mutual regulatory effect between gut microbiota and the tumor microenvironment of GBM

There is an interactive regulatory effect between gut microbiota and the tumor microenvironment (TME) of GBM, involving multiple levels such as immune regulation, metabolic regulation, neuroendocrine communication, and dietary intervention.

Immune system regulation: Some beneficial strains in the gut microbiota, such as lactobacilli and bifidobacteria (19), enhance the function of immune cells, especially the anti-tumor activity of T cells and macrophages, by producing SCFAs such as butyrate, propionate, and acetate (20). At the same time, these metabolites can promote the infiltration of immune cells in the TME, enhancing their recognition and attack ability against tumor cells (21). Imbalance of gut microbiota may lead to an increase in immunosuppressive cells, such as regulatory T cells (Tregs) and myeloid suppressive cells (MDSCs), as well as tumor associated macrophages (TAMs) leaning towards M2 type (immunosuppressive) (22). These immunosuppressive cells may weaken the anti-tumor immune response in the TME (23–25). Conversely, restoring gut microbiota balance may reduce the number or function of these immunosuppressive cells, thereby improving the TME (26).

Metabolites and signaling pathway regulation: Metabolites produced by gut microbiota can reach the brain through blood circulation, affecting the metabolism and signal transduction of GBM cells (27). For example, certain metabolites such as bile acids and indole compounds may activate specific signaling pathways within GBM cells, affecting tumor cell proliferation, migration, angiogenesis, and immune escape ability (28, 29). The chronic low-grade inflammatory state caused by imbalance of gut microbiota can lead to an increase in the levels of systemic inflammatory mediators (30), which may penetrate the brain through the blood-brain barrier (BBB), promote the sustained inflammatory response in the TME, and further exacerbate tumor progression and immune suppression (9).

The neuroendocrine communication regulation of the gut brain axis: gut microbiota can affect neurotransmitters (31). The synthesis and metabolism of aminobutyric acid, as well as the secretion of hormones such as cortisol and insulin, affect the function of neurons and glial cells in the brain through blood circulation, indirectly affecting the homeostasis of the GBM TME (16). Dysfunction of gut microbiota may affect the migration of immune cells to the brain by regulating neuroendocrine signals (32). For example, intestinal derived cytokines may stimulate the CXC ligand 12 / CXC receptor 4 (CXCL12/CXCR4) axis, promoting immune cell recruitment to the tumor site (33).

Dietary and therapeutic response: Dietary components directly affect the composition and activity of gut microbiota, thereby affecting the host’s nutritional metabolism (34). Specific dietary patterns, such as those rich in dietary fiber, antioxidants, or anti-inflammatory foods, may enhance the body’s response to chemotherapy drugs, radiation therapy, or ICI therapy by optimizing the gut microbiota, reducing therapeutic toxicity (35–37). The gut microbiota is involved in the biological transformation of drugs, including anti-tumor drugs (38). Some strains may alter the bioavailability, efficacy, or toxicity of drugs through enzymatic reactions, affecting the treatment efficacy and tolerance of patients (39).

Potential role of microbiome therapy: Fecal microbial transplantation (FMT) from healthy donors to GBM patients aims to restore gut microbiota balance, enhance anti-tumor immunity, and improve the TME (40, 41). Preliminary preclinical and clinical studies have explored its application in GBM treatment. By orally administering specific probiotics or prebiotics (such as dietary fiber, inulin, etc.) to regulate gut microbiota, promote beneficial bacterial growth, and inhibit harmful bacterial communities, it may indirectly improve the TME and overall therapeutic effect of cancer patients (42).

In depth study of these interaction mechanisms is expected to provide scientific basis for the development of new GBM treatment strategies, especially by combining existing treatment methods with gut microbiota regulation technologies.

## 4 Relationship between the gut microbiota, gut-brain axis, and GBM

The gut-brain axis comprises the bidirectional communication between the central nervous system and intestinal nervous system (43). Initially, researchers believed that the destruction of the gut microbiota would only produce pathological and physiological phenomena in the local gut, such as irritable bowel syndrome (44). However, recent studies have shown that dysbiosis of the gut microbiota not only causes pathological and physiological phenomena in the intestine but also leads to central nervous system lesions (45). Extensive preclinical studies using sterile and wild-type mice have confirmed that alterations in the gut microbiome can lead to abnormal brain signaling and behavior (46).

**Figure 4:**
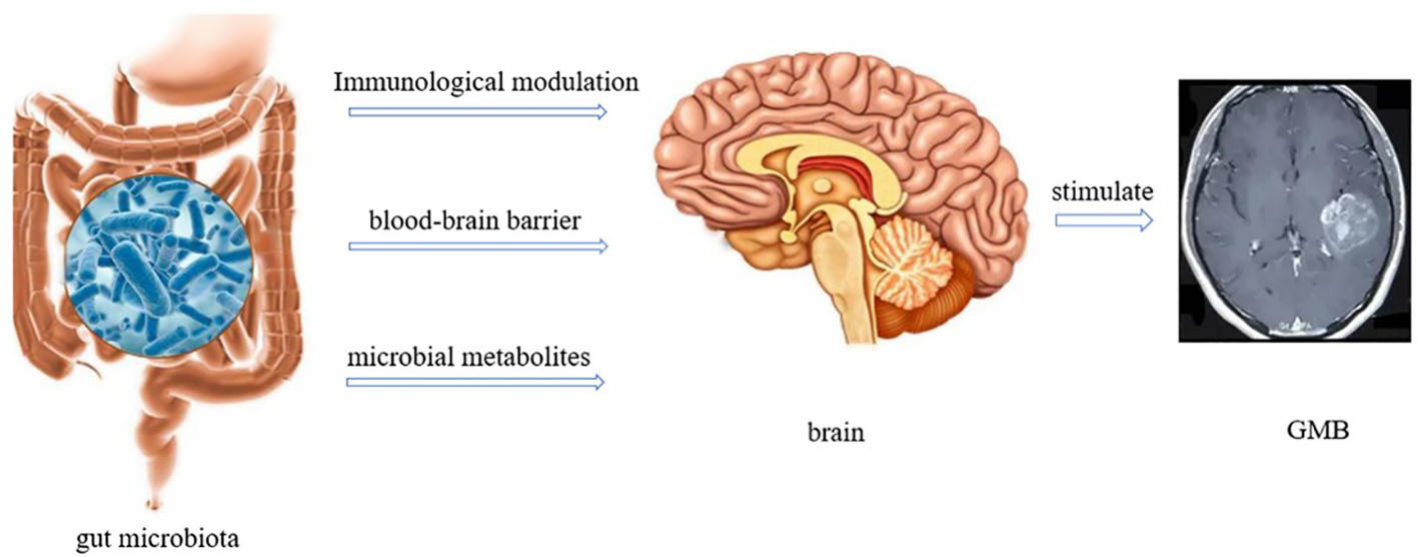
Interaction between gut microbiota and GBM (There are three ways in which gut microbiota interacts with the brain. 1) Immune regulation; 2) Directly acting through the blood-brain barrier; 3) Regulate through metabolites.).

The gut microbiota gut-brain axis refers to the interaction and influence between the gut microbiota and the brain, which plays an important role in the occurrence and treatment of GBM (16). Metabolites of the gut microbiota can act on the autonomic nervous system and neuroendocrine axis, regulating brain function and behavior. This interaction can affect the growth and development of GBM and may effect the cognition and emotions of patients (47) (**Figure 4**). The effect of the gut microbiota gut-brain axis on GBM is multifaceted. First, gut microbiota can influence GBM progression by regulating the expression of neurotransmitters. For example, gut microbiota can regulate the expression of dopamine and serotonin. However, dopamine can activate the expression of epidermal growth factor receptor (EGFR) and promote the phosphorylation of mitogen-activated protein kinase by binding to the highly expressed dopamine receptor 2 in GBM cells, thereby promoting the progression of GBM. In addition, most serotonin in the body is metabolized by the gut microbiota, and excessive secretion of 5-hydroxytryptamine can promote the proliferation of gliomas by activating the protein phosphorylation signaling pathway (48). Second, GBM disrupts the blood-brain barrier during development, allowing circulating immune cells and inflammatory mediators, such as T cells, macrophages, B cells, IL-6, IL-8, and other inflammatory mediators, to enter the brain (49). However, immune cells are inhibited in an inhibitory environment. M2 macrophages secrete IL-10, EGF, and vascular endothelial growth factor (VEGF), which inhibit T cell proliferation and promote tumor growth and angiogenesis, leading to GBM progression (50). Inflammation is an important factor that promotes GBM progression. Munoz et al. confirmed that H-Ras isoforms upregulated the expression of IL-6 and IL-8 in GBM cells, promoting their survival, invasion, and proliferation (51). Third, metabolites related to the gut microbiota, such as SCFAs, affect the function of nuclear transcription factor-κB (NF-κB) in tumor cells and immune cells. A portion of circulating SCFAs can enter the central nervous system, especially when the blood-brain barrier is disrupted. Moreover, the abnormal activation of the B pathway activates survival genes, leading to the activation of signal transducer and activator of transcription 3 (STAT3) and increased invasiveness of GBM cells (52). In addition, SCFAs can downregulate STAT1 and histone deacetylase to inhibit INF-γ, whereas Indoleamine 2, 3-dioxygenase-1 (IDO-1) can induce tumor growth factor-β (TGF-β). Helper T cell-1 (Th1) and Th2 release triggers dysregulation, which is beneficial for the M2c phenotype of microglia and inhibits cytokine production, lymphocyte proliferation, and T cell differentiation, thereby promoting tumor growth (53).

## 5 Role of the gut microbiota and gut-brain axis in ICI therapy

The interplay between gut microbiota and ICIs is extremely complex. The gut microbiota not only directly regulates the activity and function of immune cells to affect the efficacy of ICIs but also regulates the immune system of the body through the gut-brain axis, affecting ICI efficacy against tumors (54). Routy et al. found that dysbiosis of the gut microbiota can lead to tumor resistance to ICI therapy (55). FMT from ICI-responsive tumor patients into sterile or antibiotic-treated mice can improve the antitumor effect of the programmed cell death protein 1 (PD-1) blockade, whereas FMT from unresponsive patients does not show any improvement in antitumor activity (56). Importantly, the FMT response overcame the resistance of patients with melanoma to PD-1 therapy (57). A close relationship has been observed between the gut-brain axis and ICIs of tumors; however, the specific underlying mechanisms are not yet clear.

Research has shown that the gut microbiota can downregulate granulocyte-macrophage colony-stimulating factor signaling through the gut-brain axis, leading to significant expression of reactive oxygen species in activated immature myeloid cells, thereby increasing the inhibitory activity of MDSCs on T cells and enhancing ICI therapy (58). The gut microbiota promotes the development of Foxp3 Tregs and improves intestinal barrier function through the gut-brain axis, thereby inhibiting the secretion of multiple pro-inflammatory Th17 cells. FOXP3+regulatory T cells are a subset of CD4+ Th cells that serve as checkpoints for immune activation and are crucial for peripheral autoimmune prevention (59). The SCFAs produced by the gut microbiota activate cellular receptors and affect cellular metabolism. They can also enter the central nervous system through the BBB and activate protein receptors such as free fatty acid receptor 3 (FFAR3) and FFA2/FFAR2, leading to the secretion of cytokines and chemokines and the regulation of cellular programs, supporting their role as immune response enhancers and anti-inflammatory agents (60). Antigen epitope tailband measurement protein 1 and proteasome subunits in Hirae *Enterococcus* β-Type 4 tumor antigens also exhibit high similarity, which can activate CD8+ T cells and improve the effectiveness of PD-1 blockade therapy (61).

## 6 Role of the gut microbiota and gut-brain axis in regulating ICI therapy for GBM

Over the years, researchers have found that the brain has immune functions and functional lymphatic vessels, challenging the view that the brain is an immune-privileged organ (62). It was always believed that GBM is in an immunosuppressive state with poor efficacy of ICI therapy. However, Chen et al. showed that ICI therapy (anti-cytotoxic T-lymphocyte antigen 4,CTLA-4) can generate antitumor immune responses in GBM, which can produce IFN-γ. CD4+ T cells and phagocytic microglia promote the phagocytic function of microglia and enhance their effectiveness in ICI therapy. This study provides new ideas for the application of ICI therapy in GBM (63).

Recent studies have shown that the gut microbiota plays an important role in ICI therapy for GBM and that appropriate microbial activity can reduce immune suppression in glioma models and improve the response to ICI therapy. Multiple studies have confirmed that specific gut microbiota induce the function of CD8+ T cells through the gut-brain axis to enhance the efficacy of ICIs. For example, melanoma patients with a higher relative abundance of gut microbiota show increased antigen presentation, improved CD4+ and CD8+ T cell function in the peripheral blood and TME, and enhanced antitumor effects of ICIs (64). Another study showed that Firmicutes and Actinobacteria are abundant in FMT and PD-1 blockade responses. The combination of FMT and PD-1 blockade stimulates mucosal-associated invariant T and CD56+CD8+ T cells in peripheral blood mononuclear cells and upregulates the expression of human leukocyte antigen class II genes CD74 and granzyme K on CD8+ T cells at the tumor site to improve the therapeutic effect of ICIs (65).

Inosine, a metabolic product of the intestinal microbiota, can significantly enhance the ability of tumor cells to present tumor antigens. Therefore, cytotoxic immune cells can easily recognize and kill tumor cells, thereby exerting antitumor effects (66). Further mechanistic studies have shown that in inosine-treated tumor cells, IFN-γ and TNF-α significantly increased the activation of signaling pathways. The cytotoxicity of tumor-specific T and NK cells can be activated by promoting the release of perforin and granzyme, and IFN-γ can enhance antigen presentation and promote the antitumor effect mediated by inosine. Inosine also enhances the inherent immunogenicity of tumors by directly binding and inhibiting the ubiquitin-activating enzyme, ubiquitin-like modifier activating enzyme 6, rendering tumor cells sensitive to T cell-mediated cytotoxicity. Additionally, inosine can enhance the efficacy of ICIs by acting on adenosine 2A receptors on T lymphocytes (67–69).

Furthermore, SCFAs can exert multiple effects through the gut-brain axis, including the enhancement of ICIs. The SCFAs propionic acid activates the cell cycle inhibitor p21 through G protein-coupled receptor 43 and downregulates apoptosis inhibitor protein, inhibiting cancer cell proliferation, inducing cell apoptosis, and enhancing the antitumor effect of ICIs (55). SCFAs can inhibit DNA binding 2-dependent IL-12 signaling; promote the antitumor cytotoxicity of CD8 T cells; provide energy to immune cells; regulate glycolysis, the tricarboxylic acid (TCA) cycle, and fatty acid oxidation of antitumor effector cells; and improve the efficiency of ICIs (70).

## 7 Dilemma of the gut microbiota in the treatment of GBM

Basic research has shown that gut microbiota plays a certain role in the treatment of GBM, but it also faces many dilemmas, including the complexity and individual differences of gut microbiota, unclear mechanisms of action, limitations of intervention methods, drug interactions, clinical translation difficulties, drug resistance and recurrence issues, and challenges of interdisciplinary collaboration and integration (71–73).

The complexity and individual differences of gut microbiota: Gut microbiota is a complex ecosystem composed of trillions of microorganisms, and its composition and function vary from individual to individual, influenced by various factors such as genetic background, dietary habits, living environment, and disease status (71). This highly individualized characteristic poses a challenge in designing GBM treatment strategies based on gut microbiota intervention, requiring precise identification of specific bacterial species or microbiota structures that have a critical impact on treatment outcomes, and considering how to achieve personalized adjustments among different patients (12).

The mechanism of action of gut microbiota is not yet fully understood: Although previous studies have shown that gut microbiota can indirectly affect the occurrence, development, and treatment response of GBM by regulating the immune system, affecting drug metabolism, and producing bioactive substances, the specific details, upstream and downstream relationships, and causal relationships of these mechanisms need to be further elucidated. Especially in tumor ICI therapy, the interaction mechanism between gut microbiota and ICIs is complex, and their mechanisms of action in reshaping the TME and activating anti-tumor immune responses still need further research (13).

Limitations of interventions for gut microbiota: Changing the state of gut microbiota to assist GBM treatment typically involves methods such as prebiotics, probiotics, synbiotics, and FMT. However, the evidence for the effectiveness and safety of these methods in GBM patients is not yet sufficient. The colonization effect and long-term stability of probiotics are uncertain. The selection and dosage of prebiotics require precise regulation. The effectiveness of synbiotics depends on the synergistic effect of multiple components and is difficult to standardize. FMT may face difficulties in donor screening, suboptimal post-transplant bacterial colonization, and potential risks of disease transmission. In addition, the dynamic changes in gut microbiota may lead to unstable intervention effects (41–43).

Interaction between gut microbiota and drugs: The gut microbiota can metabolize certain chemotherapy drugs and targeted drugs, changing their *in vivo* concentration, bioavailability, and toxicity (74, 75). The efficacy and toxicity of chemotherapy drugs such as temozolomide may be influenced by gut microbiota metabolism (76). However, predicting and regulating these interactions to optimize drug efficacy and reduce side effects remains challenging, especially in GBM where patients typically require multiple drug combinations, increasing the complexity of drug microbiota interactions (77).

Clinical transformation challenges of gut microbiota: Although basic research has revealed the potential value of gut microbiota in GBM, translating these findings into clinical practice still faces many challenges. This includes the need to design rigorous clinical trials to validate the effectiveness and safety of gut microbiota interventions, establish standardized methods for gut microbiota detection and analysis, and address ethical, regulatory, and patient acceptance issues.

Drug resistance and recurrence of GBM: GBM has the characteristics of high invasiveness, easy recurrence, and tolerance to traditional treatment methods. Although the gut microbiota may affect the response of tumors to treatment, it is currently unclear how to effectively overcome the therapeutic resistance of GBM or prevent and delay tumor recurrence by regulating the microbiota (78).

Multidisciplinary collaboration and integration: The interdisciplinary field of gut microbiota and GBM involves multiple disciplines such as oncology, immunology, microbiology, pharmacology, etc., requiring close collaboration among interdisciplin-ary experts to jointly promote research progress and clinical applications. However, in practical operation, there may be difficulties in disciplinary barriers, allocation of research resources, and standardization of research methods.

Based on the above difficulties, continuous scientific research, technological innovation, and in-depth interdisciplinary cooperation are needed to solve these problems, in order to truly bring survival benefits to patients with GBM through gut microbiota.

## 8 Summary and perspective

The gut microbiota has a significant effect on ICI therapy for GBM. The gut microbiota regulates the function, metabolism, and inflammatory response of the immune system through the gut-brain axis, affecting the efficacy and safety of ICIs for GBM. First, the gut microbiota can affect the function of the immune system, thereby affecting ICI therapy for GBM. The intestinal microbiota enhances the immune system by stimulating the activation and proliferation of immune cells, thereby improving the efficacy of ICI therapy. Second, the gut microbiota can affect the immune microenvironment of GBM by regulating the infiltration and activation of immune cells, thereby affecting the efficacy of ICIs. The immune microenvironment refers to the composition and state of immune cells, the matrix, and cytokines around tumors, which play an important regulatory role in tumor growth and spread. Third, gut microbiota can affect the nutritional and metabolic status of patients with GBM, thereby affecting the efficacy of ICI therapy. Intestinal microbiota participate in nutritional and metabolic processes in the human body, including vitamin synthesis and amino acid metabolism. The nutritional and metabolic statuses of patients can be improved by regulating the composition and activity of the gut microbiota, thereby enhancing the therapeutic effects of ICIs.

The regulation of the gut microbiota through the gut-brain axis in ICI therapy for GBM is a complex process that requires further research to gain a deeper understanding of its specific mechanisms and effects. Currently, relatively little research is available on the mechanism and efficacy of the gut microbiota in GBM, and further research is required to verify its specific effects and potential therapeutic value. Simultaneously, neural therapy targeting the gut microbiota must consider individual differences and safety issues.

## Author contributions

HZ: Resources, Writing – original draft. YH: Resources, Writing – original draft. TW: Data curation, Writing – original draft. EB: Data curation, Resources, Writing – review & editing. SL: Supervision, Writing – review & editing. LH: Resources, Writing – original draft. TX: Data curation, Funding acquisition, Supervision, Writing – review & editing.
